# Berberine Promotes Cardiac Function by Upregulating PINK1/Parkin-Mediated Mitophagy in Heart Failure

**DOI:** 10.3389/fphys.2020.565751

**Published:** 2020-09-25

**Authors:** Miyesaier Abudureyimu, Wenjun Yu, Richard Yang Cao, Yingmei Zhang, Haibo Liu, Hongchao Zheng

**Affiliations:** ^1^Cardiovascular Department, Shanghai Xuhui Central Hospital, Zhongshan-Xuhui Hospital, Fudan University, Shanghai, China; ^2^Shanghai Institute of Cardiovascular Diseases, Zhongshan Hospital, Fudan University, Shanghai, China; ^3^Department of Cardiology, Qingpu Branch of Zhongshan Hospital, Fudan University, Shanghai, China

**Keywords:** PINK1, mitophagy, berberine, cardiac hypertrophy, heart failure

## Abstract

Berberine has been verified to protect cardiac function in patients with heart failure (HF). However, the mechanism(s) involved in berberine-mediated cardioprotective effects has not been clearly elucidated. The aim of this study was to further investigate the mechanism(s) involved in the beneficial effects of berberine on transverse aortic contraction (TAC)-induced chronic HF. Mice were randomly divided into four groups. Berberine was administered at a dose of 50 mg/kg/day for 4 weeks via oral gavage. Our findings showed that TAC-induced pressure overload (PO) prompted cardiac dysfunction, cardiac hypertrophy, interstitial fibrosis, cardiomyocyte apoptosis and mitochondrial injury, accompanied with suppressed mitophagy, the effects of which were attenuated by berberine. Furthermore, mitophagy regulators PINK1 and mito-Parkin were downregulated in TAC-induced HF, while berberine upregulated PINK1/Parkin-mediated mitophagy. Notably, knockdown of PINK1 by small interfering RNA significantly suppressed Parkin-mediated mitochondrial ubiquitination and nullified the beneficial actions on HF exerted by berberine. Taken together, our results indicated that berberine plays a critical role in attenuating cardiac hypertrophy and preserving cardiac function from PO induced HF. The potential underlying mechanism is the activation of mitochondrial autophagy via PINK1/Parkin/Ubiquitination pathway.

## Introduction

Heart failure (HF) is considered as a clinical syndrome, in which structural, neurohumoral, cellular, and molecular mechanisms are activated after cardiac injury (Van der Meer et al., 2019). HF imposes a great health threat globally as American Heart Association (AHA) in 2017 predicted more than 8 million adults to be afflicted with HF by 2030 (Stuart, 2014). Although a remarkable progress has been made with the development of a series of therapies including drug therapy, interventional therapy, surgical treatment and effective management, it is still not fully satisfied with the prognosis of HF (Hoffman, 2016). Therefore, more work needs to be taken to further clarify the pathogenesis of HF and to search for new therapy target.

Berberine (BBR) is an isoquinoline alkaloid extracted from *Berberidaceae* and also a basic component of the Chinese herb called coptis (Wang et al., 2017). Berberine has been used for anti-infection of various parasitic and fungal, also for anti-diarrheal (Xia and Luo, 2015). Ample clinical trials and animal studies have demonstrated a variety of cardiovascular pharmacological activity of BBR, such as antihypertension, antiarrhythmia, anti-myocardial remodeling and dyslipidemia regulation (Kumar et al., 2015; Xia and Luo, 2015; Imenshahidi and Hosseinzadeh, 2019). An experimental study demonstrated that berberine could prevent left ventricular hypertrophy induced by pressure-overload (PO) with undefined mechanism (Hong et al., 2002).

Mitochondria are essential for energy generation to maintain cardiac contraction (Shirakabe et al., 2016). Effective degradation and removal of dysfunctional mitochondria is crucial for cardiomyocyte survival (Sciarretta et al., 2011; Lavandero et al., 2015). Mitochondrial quality control is important for cellular energy supply and physiological processes (Tong et al., 2019). The current experimental studies have suggested a potential effect of berberine on regulating mitochondrial dynamic homeostasis (Gomes et al., 2012; Yan et al., 2017; Xu et al., 2018). Autophagy is a critical physiological process for degradation of senescent and damaged protein, while selective autophagic degradation of dysfunctional mitochondria is termed as mitophagy (Mizushima, 2007; Pedrozo et al., 2013; Frati et al., 2018). Berberine alleviates cardiac ischemia/reperfusion injury by inhibiting excessive autophagy in cardiomyocytes (Qing et al., 2018). Cardiac hypertrophy is a maladaptive change in response to PO, and is also an important risk factor for further development to HF. Berberine could attenuate cardiac hypertrophy associated with enhanced autophagy via inhibiting activation the mechanism of the mammalian target of rapamycin (mTOR), extracellular signal-regulated kinase (ERK1/2), and p38 mitogen-activated protein kinase (MAPK) phosphorylation (Li et al., 2014). Although autophagy has an important role in berberine-mediated cardioprotective effects on cardiac hypertrophy, the role of mitophagy in berberine-elicited beneficial actions on pathological hypertrophy and HF remains unknown.

Mitophagy, the selective removal of damaged mitochondria, is a fundamental process for mitochondrial quality control (Nah et al., 2017; Thai et al., 2019). Mitophagy is activated the early stage of cardiac hypertrophy during HF, which ultimately promotes cell apoptosis (Saito and Sadoshima, 2015). Mitophagy is reported to be regulated mainly by PTEN-induced putative kinase 1 (PINK1)/ cytosolic E3 ubiquitin ligase (Parkin). PINK1 and Parkin are the two key molecules involved in regulation of mitophagy. Depolarization of mitochondria blocked mitochondrial import of PINK1, leading to PINK1 accumulation on the mitochondrial membrane, which then phosphorylates and recruits Parkin to the outer mitochondrial membrane (OMM) from the cytosol by phosphorylating mitofusin 2 (MFN2). The translocation of Parkin onto mitochondria triggered the ubiquitin-dependent mitophagic clearance of damaged mitochondria (Gladkova et al., 2018). The protein levels of PINK1 and mitochondrial Parkin were decreased during the progression of HF induced by transverse aortic contraction (TAC) surgery in murine model, consistent with the changes of mitophagy activity (Frati et al., 2018). However, whether consistent pathway is involved in berberine-mediated mitophagy regulation, if any, remains to be elucidated.

Therefore, in the current study, berberine was administrated after TAC surgery induced chronic HF mouse model. We assumed that berberine can greatly improve cardiac function in mouse hearts. Using *in vitro* and *in vivo* studies, verified that berberine inhibited of excessive autophagy, also revealed the possible mechanism involved in cardiac protection. Therefore, our data implicate that berberine might be a promising therapeutic agent to improve cardiac function from chronic HF.

## Materials and Methods

### Animals

All the protocols of *in vivo* animal experiments were approved by the Institutional Animal Care and Use Committee of the Zhongshan Hospital Fudan University (Shanghai, China) in compliance with the Guide for the Care and Use of Laboratory Animals (NIH Publication No. 85-23, revised 1996). Eight to ten weeks male C57BL/6J wild-type mice were commercially obtained from Shanghai Model Organism Center, Inc. After arrival, all mice were carefully raised following regular circadian cycle with enough sterilized water and food before and after TAC surgery.

### Experimental Design and TAC Surgery

After acclimatization for at least 3 days, chronic HF model was induced by TAC surgery, which was described previously (Tavakoli et al., 2017). Briefly, mice were anesthetized with pentobarbital sodium (30–45 mg/kg, intraperitoneal, Sigma-Aldrich, St. Louis, MO, United States). A midline cervical incision was performed to expose the aortic arch and to isolate the vessel. The fraction of the aorta localized between the brachiocephalic artery and left common carotid artery was constricted by a 27G needle and a 6-0 silk thread. After closing the incision, mice were subcutaneously injected with penicillin G (10000 U) for infection prevention. Sham-operation was performed in age-matched mice with the same procedure but without aorta banding.

After successful surgery, mice were randomly divided into four groups: sham-operated mice with vehicle (Sham-Veh group), sham-operated mice with berberine (Sham-BBR group), TAC-operated mice (TAC-Veh group), TAC-operated mice with berberine (TAC-BBR group). Berberine (Sigma-Aldrich) dissolved in 5% sterilized glucose was administered immediately after TAC surgery at a dose of 50 mg/kg/d for 4 weeks via oral gavage. Sham-operated mice were received same volume of 5% sterilized glucose (vehicle) routinely. Four weeks after surgery, all surviving mice were assessed by echocardiography and were sacrificed for histological analysis and mechanism exploration.

### Isolation and Culture of Primary Neonatal or Adult Mouse Cardiomyocytes (NMCMs or AMCMs)

For isolation of NMCMs (Ehler et al., 2013), sterilized neonatal mouse hearts were rapidly excised and quickly minced into fragments followed by enzymatic digestion with a collagenase mixture (Worthington, Lakewood, NJ, United States). After digestion for several minutes, the fragments were placed in a sterilized culture dish to sediment for about 1 min. Then the supernatant containing a great quantity of cardiomyocytes was replanted in another collagen-coated dish at approximately 1.5 × 10^5^ cells/cm^2^. After 48 h for static incubation, the culture media was refreshed with complete DMEM (Gibco, Grand Island, NY, United States) containing 4500 mg/L glucose, 4 mM L-glutamine, 110 mg/L sodium pyruvate, 1% (v/v) penicillin/streptomycin (Gibco) and 10% (v/v) fetal bovine serum (FBS, Gibco). Cells were cultured in a 37°C incubator containing 95% air and 5% CO_2_.

For isolation of AMCMs (Ackers-Johnson et al., 2016), mice were anesthetized, and the chest was quickly incised to fully expose the heart. After cutting the descending aorta, the heart was immediately flushed by injection of 7 mL EDTA buffer (Gibco) into the right ventricle (RV). After that, heart was transferred to a 60-mm dish with fresh EDTA buffer. Digestion was achieved by sequential injection of 10 mL EDTA buffer, 3 mL perfusion buffer, and 3050 mL collagenase buffer into the left ventricle (LV). Atria, LV, and RV were separated and gently pulled into 1-mm pieces. Cellular dissociation was completed by gentle trituration, and digestion was stopped by 5 mL stop buffer. After passing through a 100-μm filter and receiving 4 sequential rounds of gravity settling, 3 intermediate calcium reintroduction buffers were used to gradually restore calcium concentration to physiological levels. A yield of at least 80% rod-shaped AMCMs were deemed successful. Where required, cells were plated onto laminin (5 μg/mL) precoated dishes at an appropriate density in an incubator (37°C, 5% CO_2_). After 1 h, and media was refreshed every 48 h by prewarmed culture medium.

*In vitro* HF model was simulated by phenylephedrine (PE, Sigma-Aldrich, dissolved in DMSO, 100 μM for 24 h). Berberine was dissolved in DMSO and administrated at the concentration of 5 μM for 24 h. Inhibition of autophagy was induced by inhibitors including 3-MA (Sigma-Aldrich, 10 mM for 2 h) or bafilomycin A1 (Baf, Sigma-Aldrich, 100 nM for 2 h).

### Echocardiographic Examination

Four weeks after TAC surgery, mice were inhalation anesthetized with a mixture of 2% isoflurane and oxygen. Systolic cardiac function was evaluated across thoracic region using 2-D guided M-mode echocardiography (Visual Sonics Vevo 2100, Toronto, Canada) equipped with a 15–16 MHz linear transducer. M-mode echocardiograms of LV were recorded from the parasternal long-axis view. Left ventricular ejection fraction (LVEF) and fractional shortening (LVFS) were calculated. LVEF was calculated from left ventricular end-diastolic volume (LVEDV) and left ventricular end-systolic volume (LVESV) by the equation of (LVEDV-LVESV)/LVEDV × 100%, whereas LVFS was calculated from left ventricular end-diastolic diameter (LVEDD) and left ventricular end-systolic diameter (LVESD) by the equation of (LVEDD-LVESD)/LVEDD × 100%.

### Histological Staining

To determine the size of the heart, whole hearts were firstly fixed in 4% paraformaldehyde and photographed by a digital camera with a ruler beside. Then the fixed hearts were cut longitudinally, embedded in paraffin and sliced into 4-μm thick sections for hematoxylin-eosin (H&E) staining, Masson Trichrome staining and wheat germ agglutinin (WGA) staining as described previously (Zhang et al., 2017). The interstitial fibrosis of each slice was assessed by using Image J software (National Institute of Health, Version 1.8). Cross-sectional area of each cardiomyocyte was measured by Image J and presented in square millimeters.

### TUNEL Assay

Apoptosis of myocardium and primary cardiomyocytes were determined by terminal deoxynucleotidyl transferase-mediated dUTP-Xnick end labeling (TUNEL, Roche, Indianapolis, IN, United States) staining. Micrographs of TUNEL-positive and DAPI-stained nuclei were captured randomly using a fluorescence microscope or a confocal microscope (Leica, Germany) and were counted using the Image J software. Apoptotic cells were calculated as the percentage of TUNEL-positive cells among total nuclei. Nine microscopic fields with at least 100 cells per group were randomly selected to assess apoptosis.

### Transmission Electron Microscopy (TEM)

Murine hearts were cut into pieces and fixed in 2% glutaraldehyde for at least 24 h. After immersed in 2% osmium tetroxide and 1% aqueous uranyl acetate for 1 h, and washed with a series of ethanol solutions (50, 70, 90, and 100%), tissues were transferred to propylene oxide, incubated in a mixture of propylene oxide and EMbed 812 for 1 h and then polymerized at 70°C. Sections were cut into 75–80 nm using a Leica ultramicrotome equipped with a Diatome diamond knife and collected onto 200-mesh copper grids. Following poststained in 5% uranyl acetate for 10 min and in Reynold’s lead citrate for 5 min, sections were observed under a 40–120 kV transmission electron microscope (Hitachi H600 Electron Microscope, Hitachi, Japan).

### Reactive Oxygen Species (ROS)

Living AMCMs were rinsed with 37°C PBS and incubated with 10 μM DCFH-DA fluorescence probe (Beyotime Biotechnology, Shanghai, China, 1:1000, no serum) for 20 min at 37°C. Cells were observed using a Leica confocal microscope. ROS fluorescence intensity from n fields per group was evaluated by Image J.

### Mitochondrial Membrane Potential (MMP)

MMP was determined by JC-1 staining (Beyotime Biotechnology). Living NMCMs were rinsed with 1 × PBS and incubated with 5 μM JC-1 dye at 37°C for 20 min. Cells were visualized using a Leica confocal microscope. Mitochondria with higher MMP showed red fluorescence (J-aggregate), while those with MMP loss showed green fluorescence (J-monomer). MMP was calculated as red fluorescence/green fluorescence by Image J.

### Immunofluorescence Assay

Adult mouse cardiomyocytes were fixed and permeabilized at room temperature. AMCMs were incubated with mouse anti-COXIV antibody (Cell Signaling Technology, Danvers, MA, United States, 1:200) and rabbit anti-LC3 antibody (Abcam, Cambridge, MA, United States, 1:200) overnight at 4°C following blocking for 1 h. Subsequently, AMCMs were incubated with goat anti-rabbit Alexa Fluor 488 (Cell Signaling Technology, 1:1000) and goat anti-mouse Alexa Fluor 546 (Cell Signaling Technology, 1:1000) for 1 h. Immunofluorescence was assessed on a Leica confocal microscope with a 63 × oil objective.

### Mitochondrial Isolation

Neonatal mouse cardiomyocytes mitochondria were isolated using Abcam’s benchtop mitochondria isolation kit. After frozen and thawed to weaken membranes, cells were suspended in Reagent A at 5 mg/mL on ice for 10 min. Then cells were homogenized using a glass Dounce homogenizer, and centrifuged at 1000 × *g* for 10 min at 4°C twice. The supernatant was then centrifuged at 12000 × *g* for 10 min at 4°C. The remaining pellet was resuspended in Reagent C with phosphatase/protease inhibitor and frozen at −80°C. Mitochondrial protein concentration was detected using Enhanced BCA Protein Assay Kit (Beyotime Biotechnology).

### Protein Extraction and Western Blot Analysis

The methods used for protein extraction from *in vitro* and *in vivo* samples and for western blot analysis have been described previously (Yu et al., 2017). The antibodies used include BNP (Abcam, ab236101, 1:500), MHC (Abcam, ab180779, 1:1000), Vinculin (Abcam, ab129002, 1:500), p62 (Cell Signaling Technology, #5114, 1:1000), LC3 (Abcam, ab48394, 1:500), β-actin-HRP (Kang Cheng, KC-5A08, 1:5000), VDAC1 (Abcam, ab154856, 1:1000), Ubiquitin (PTM BIO, PTM-1107, 1:2000), PINK1 (Abcam, ab23707, 1:500), Parkin (Abcam, ab77924, 1:500), FUNDC1 (Abcam, ab224722, 1:500), PHB2 (Santa Cruz, sc133094, 1:1000), BNIP3L (Cell Signaling Technology, #12396, 1:1000), p-Parkin (Ser65) (Affinity Biotech, AF3500, 1:1000).

### Statistical Analysis

Data were expressed as Mean ± SEM for the indicated number of experiments. The difference was evaluated using one-way ANOVA followed with the Tukey *post hoc* test (Graph-Pad 8.0, Graph Pad Software, La Jolla, CA, United States). *P*-values of < 0.05 were considered statistically significant.

## Results

### Berberine Prevents TAC-Induced Cardiac Dysfunction

In order to evaluate the safe dose of berberine with different administration, C57BL/6J mice were treated with two different operations, oral administration and intraperitoneal injection, respectively. Five doses of berberine per day (0, 10, 50, 75, and 100 mg/kg) were set for oral administration, while five doses of berberine per day (0, 5, 7.5, 10, and 20 mg/kg) for intraperitoneal injection. As shown in Supplementary Figure 1, oral administration showed safer compared to intraperitoneal injection group prominently. Notably, all of mice treated with 50 mg/kg/d berberine survived after 4 weeks by oral administration, which was selected as the recommended dosage during the following experiment.

**FIGURE 1:**
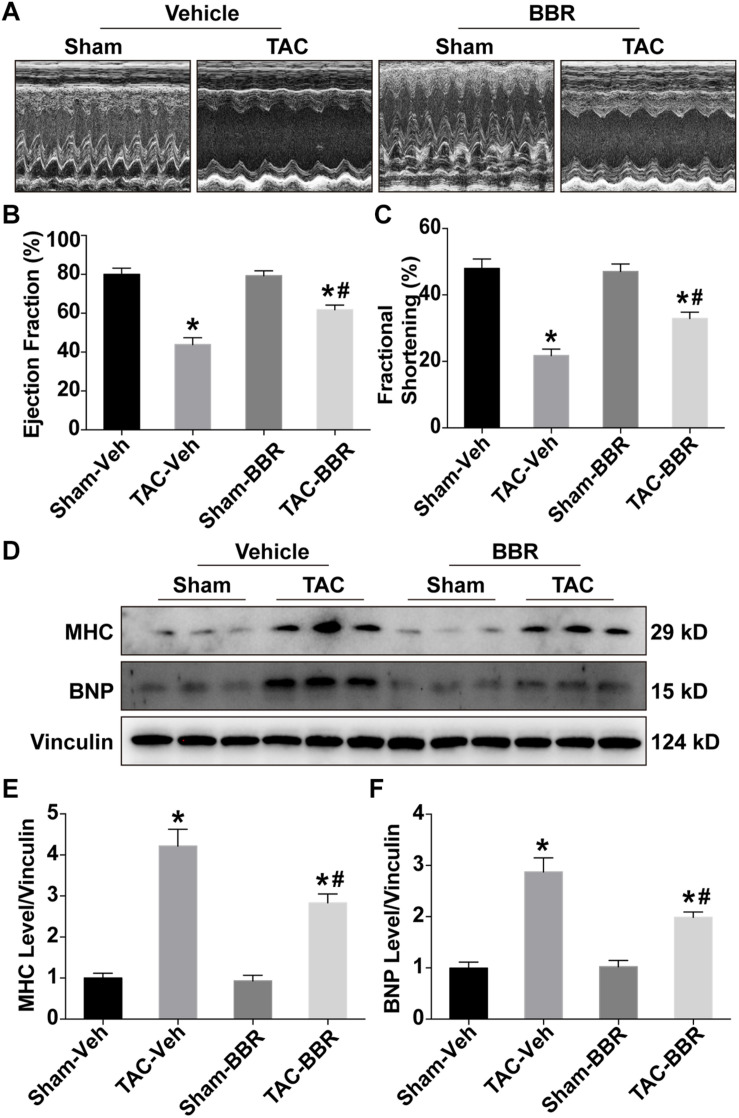
Echocardiographic properties and expression of heart failure makers in Sham- and TAC-operated mice treated with or without berberine (BBR). **(A)** Representative echocardiographic images (*n* = 5–6); **(B)** Ejection fraction (%); **(C)** Fractional shortening (%); **(D–F)** Expression of heart failure maker brain natriuretic peptide (BNP) and myosin heavy chain (MHC) by western blot (*n* = 3). Mean ± SEM, *p < 0.05 vs. Sham-Veh group; # *p* < 0.05 vs. TAC-Veh group.

To evaluate the effect of berberine on TAC-induced changes in the cardiac systolic function, transthoracic echocardiography was performed 4 weeks after TAC surgery. Our results showed that both LVEF and LVFS in the TAC group were significantly decreased as compared with the sham group, the effect of which were significantly attenuated in the TAC-BBR group (Figures 1A–C). However, there was no significant difference between sham and berberine group concerning both two parameters. Western blot showed that compared with the sham group, TAC induced an overt increase of brain natriuretic peptide (BNP) and myosin heavy chain (MHC), markers indicating HF, the effect of which was abrogated by berberine treatment (Figures 1D–F). These data indicate that berberine can preserve cardiac systolic function after TAC operation.

### Berberine Prevents TAC-Induced Cardiac Hypertrophy

**FIGURE 2:**
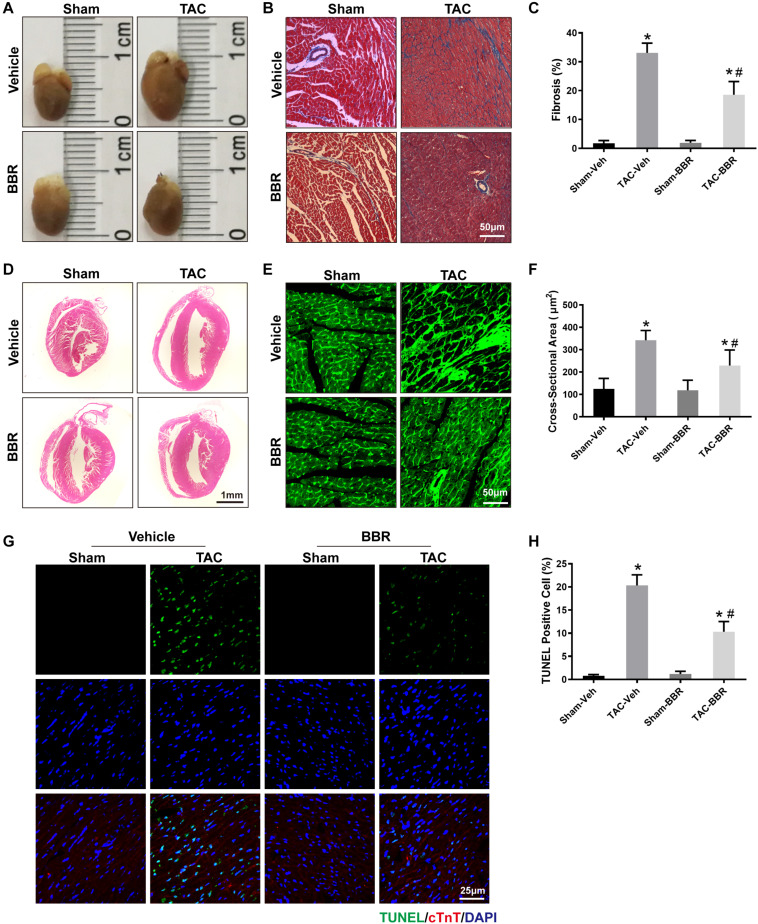
Properties of myocardial damage in Sham- and TAC-operated mice treated with or without BBR. **(A)** Representative images of the whole heart; **(B)** Representative images of Masson’s Trichrome staining (Scale bars = 50 μm); **(C)** Quantified data of interstitial fibrosis for each group (*n* = 10–21 fields);**(D)** Representative images of H&E staining (Scale bars = 1 mm); **(E)** Representative wheat germ agglutinin (WGA) staining (Scale bars = 50 μm); **(F)** WGA quantification of cardiomyocyte cross-sectional area (*n* = 350–1000 cells); **(G)** Representative TUNEL/DAPI staining images (Scale bars = 25 μm); **(H)** Quantified data of myocardial apoptosis for each group (*n* = 6). Mean ± SEM, * *p* < 0.05 vs. Sham-Veh group; # *p* < 0.05 vs. TAC-Veh group.

Cardiac hypertrophy is not only a maladaptive change in response to PO, but also an important risk factor to further develop HF. To evaluate the effect of berberine on TAC-induced cardiac hypertrophy, cardiac morphology including the size of the heart, H&E staining, Masson’s Trichrome staining and WGA staining were applied. Our data showed that hearts were enlarged in response to 4-week TAC compared to sham-operated group. Treatment with berberine, however, attenuated the enlargement of hearts induced by 4-week TAC surgery (Figure 2A). The parameter was consistent with the H&E -stained histology sections showing that berberine reduced the size of the left ventricle which was larger 4 weeks after TAC surgery (Figure 2D). Masson’s Trichrome staining showed that TAC surgery induced obvious cardiac interstitial fibrosis which was attenuated by administration of berberine (Figures 2B,C). WGA staining showed that the cross-sectional area of myocardium was significantly increased after TAC surgery, which was alleviated by treatment with berberine (Figures 2E,F). These data indicate that TAC-induced cardiac hypertrophy was significantly ameliorated by berberine administration.

### Berberine Ameliorates TAC-Induced Cardiomyocyte Apoptosis and Mitochondrial Dysfunction

**FIGURE 3:**
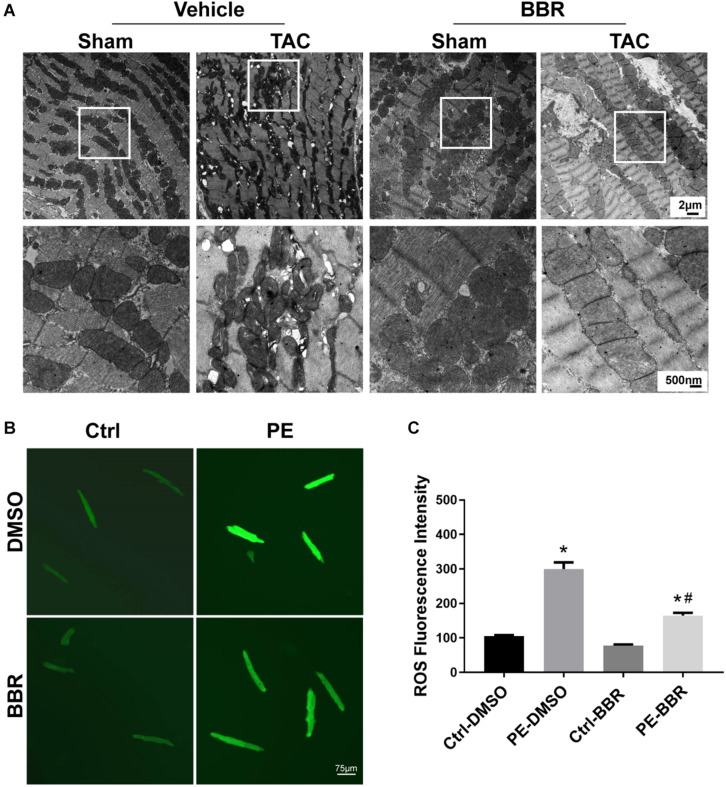
Determination of mitochondrial properties in Sham- and TAC-operated mice treated with or without BBR. **(A)** Representative mitochondrial morphology by transmission electron microscopy (TEM); **(B,C)** Representative images and quantified data of reactive oxygen species (ROS) for each group (*n* = 7). The squares indicate impaired mitochondrion. Scale bars, 2 μm, 500 nm and 75 μm (TEM). Mean ± SEM, * *p* < 0.05 vs. Ctrl-DMSO group; # *p* < 0.05 vs. PE-DMSO group.

To further determine the effect of berberine on TAC-induced cardiomyocyte injury, TUNEL/DAPI staining was applied to evaluate the ratio of apoptotic cells to total cardiomyocytes. Our data showed that there is a significant rise of apoptotic cells in failing hearts induced by 4-week TAC, the effect of which was overtly suppressed by berberine (Figures 2G,H). As mitochondria function plays a critical role in TAC-induced cardiac hypertrophy and HF, mitochondrial morphology changes and function were evaluated by transmission electron microscopy (TEM) and reactive oxygen species (ROS) detection 4 weeks after TAC surgery. TEM revealed more disorganized, swollen and damaged mitochondria in heart with TAC treatment. Berberine administration significantly attenuated the injured changes of mitochondria induced by TAC-mediated PO (Figure 3A). To further evaluated the function of mitochondria, AMCMs were isolated and mitochondrial function was evaluated by ROS detection. Results showed that significant ROS accumulation was observed in AMCMs isolated from TAC-induced failing hearts. Berberine blocked the TAC-induced elevation in ROS production in AMCMs (Figures 3B,C). These data indicate that berberine contributes to the improvement of cell apoptosis and mitochondrial dysfunction induced by TAC-mediated PO.

### Berberine Restores the Level of Autophagy and Mitophagy After TAC

**FIGURE 4:**
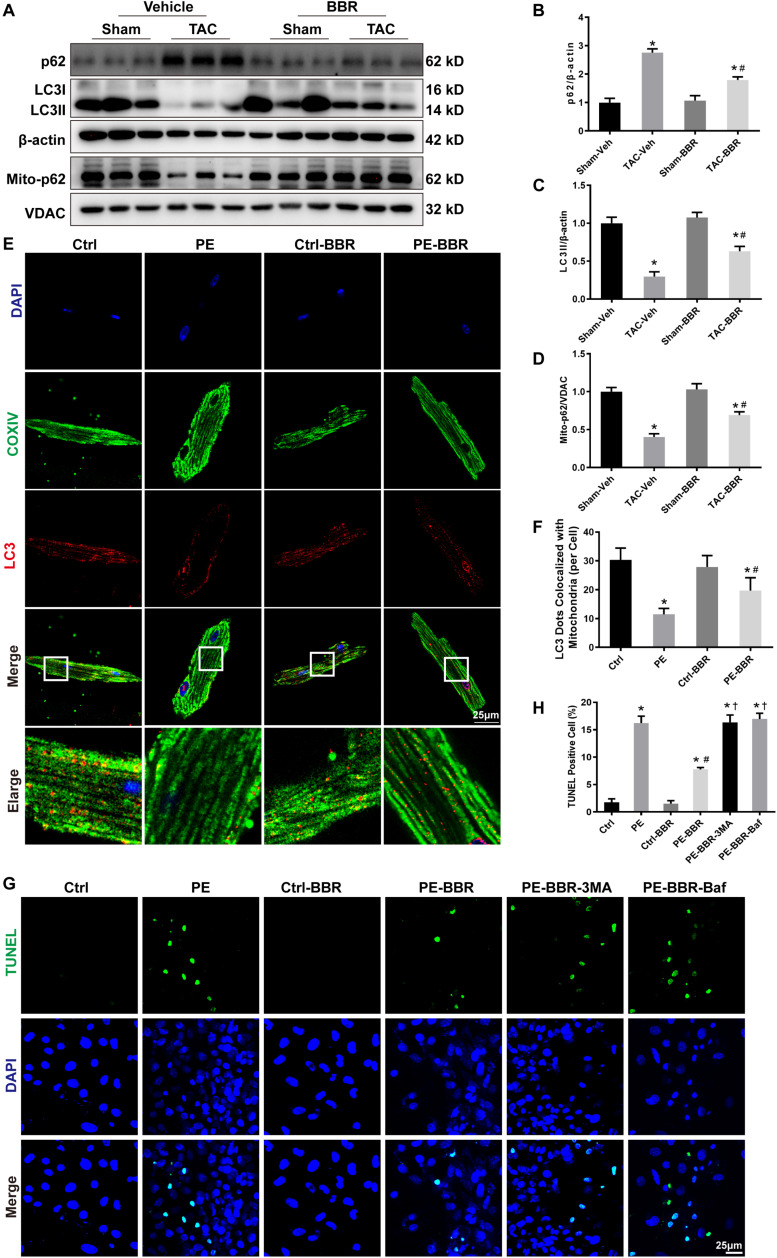
Determination of autophagic properties in Sham- and TAC- operated mice treated with or without BBR. NMCMs were treated with or without PE in the presence or absence of the autophagy inhibitor 3-MA (10 mM for 2 h) and Bafilomycin A (100 nM for 2 h). **(A)** Representative gel blots for each group by western blot (*n* = 3); **(B)** p62 to β-actin ratio; **(C)** LC3II to β-actin ratio; **(D)** Mito-p62 to VDAC ratio, Mean ± SEM, * *p* < 0.05 vs. Sham-Veh group; # *p* < 0.05 vs. TAC-Veh group; **(E)** Representative LC3/COXIV/DAPI staining images for each group (Scale bar = 25 μm); **(F)** Quantified data of LC3 dots colocalized with mitochondria (COXIV) for each cell (*n* = 9), Mean ± SEM, * *p* < 0.05 vs. Ctrl group; # *p* < 0.05 vs. PE group; **(G)** Representative TUNEL/DAPI staining images for each group (Scale bar = 25 μm); **(H)** Quantified data of cardiomyocytes apoptosis for each group (*n* = 6). Mean ± SEM, * *p* < 0.05 vs. Ctrl group; # *p* < 0.05 vs. PE group. † *p* < 0.05 vs. PE-BBR group.

Mitophagy is important for mitochondrial quality control. To elucidate whether the aforementioned effect of berberine is mediated through mitophagy, western blot was carried out to measure autophagy associated biomarkers light chain 3 (LC3) and p62. Data showed that LC3 II protein level was significantly decreased after TAC surgery while p62 level was obviously increased following 4-week TAC. Berberine treatment overtly attenuated the changes of LC3II and p62 four weeks after TAC operation (Figures 4A–C). Meanwhile, mitochondrial p62 was reduced after 4-week TAC while elevated by berberine (Figures 4A,D). In addition, to further reveal the effect of berberine on mitophagy, immunofluorescence was applied to evaluate co-localization between LC3 (red fluorescence) and mitochondria (COXIV, green fluorescence). This study revealed overtly decreased number of LC3 dots co-localized with mitochondria after 4-week TAC, the effect of which was ameliorated by berberine (Figures 4E,F). Furthermore, we evaluated the effect of berberine -mediated mitophagy on HF-induced apoptosis by TUNEL assay. Data showed that PE treatment induced significant increase of apoptotic cardiomyocytes while berberine supplementation obviously decreased apoptotic numbers in PE-treated cardiomyocytes, which was further markedly reversed by either treatment with autophagy inhibitor 3-MA or bafilomycin A1 (Figures 4G,H).

### Signaling Mechanism(s) in Berberine-Mediated Mitophagy Regulation in TAC-Induced Heart Failure

**FIGURE 5:**
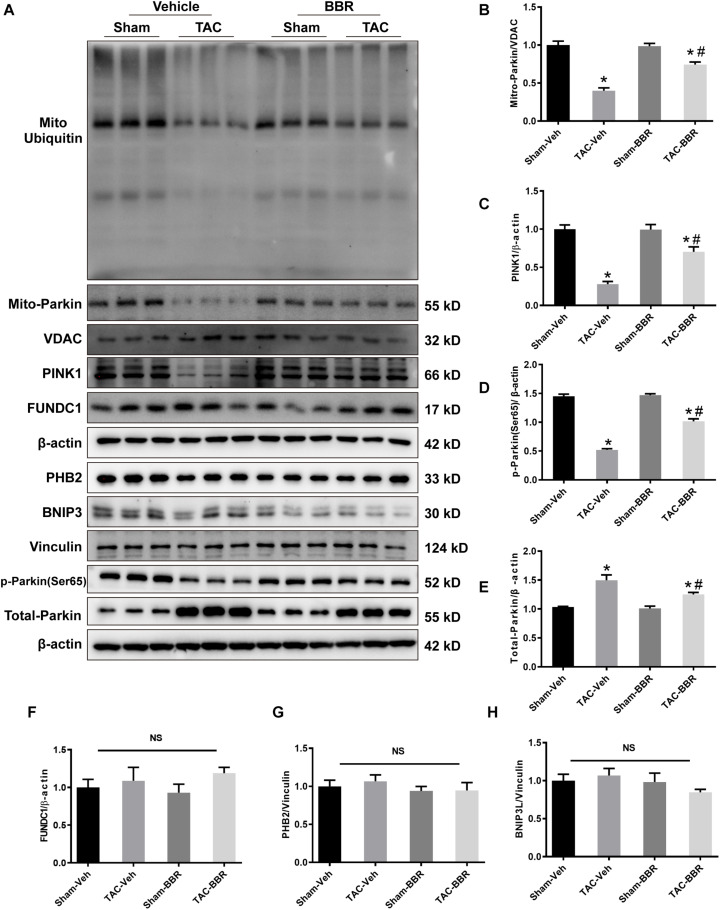
Possible mechanism(s) underlying in the mitophagic regulation of berberine in Sham- and TAC-operated mice treated with or without BBR. **(A)** Representative gel blots for each group by western blot (*n* = 3); **(B)** Mito-Parkin to VDAC ratio; **(C)** PINK1 to β-actin ratio; **(D)** p-Parkin(ser65) to β-actin ratio; **(E)** Total-Parkin to β-actin ratio; **(F)** FUNDC1 to β-actin ratio; **(G)** PHB2 to Vinculin ratio; **(H)** BNIP3L to Vinculin ratio. Mean ± SEM, * *p* < 0.05 vs. Sham-Veh group; # *p* < 0.05 vs. TAC-Veh group.

To determine the mechanism(s) underlying in the mitophagic regulation of berberine in TAC-induced HF, we examined the activity of several mitophagy pathways (PINK1/Parkin- and mitophagy receptors-dependent) by western blot. There was no difference of FUN14 domain containing 1 (FUNDC1), prohibitin-2 (PHB2) and BCL2 interacting protein 3 like (BNIP3L) in all the four groups (Figures 5A,F–H). Intriguingly, PINK1 protein level was decreased after 4-week TAC surgery. Total Parkin was increased while mitochondrial Parkin was decreased combined with reduced mitochondrial ubiquitin after TAC-induced PO. These data indicated that PINK1-Parkin-ubiquitin pathway was depressed during TAC-induced HF. However, the depression was partially reversed by berberine treatment (Figures 5A–E). Our findings suggest that berberine plays a protective role in mouse model of HF possibly by modulating the PINK1/Parkin/Ubiquitin-mediated mitophagy process.

### The Protective Effects of Berberine Against HF Are Abolished in the Presence of PINK1 Downregulation

**FIGURE 6:**
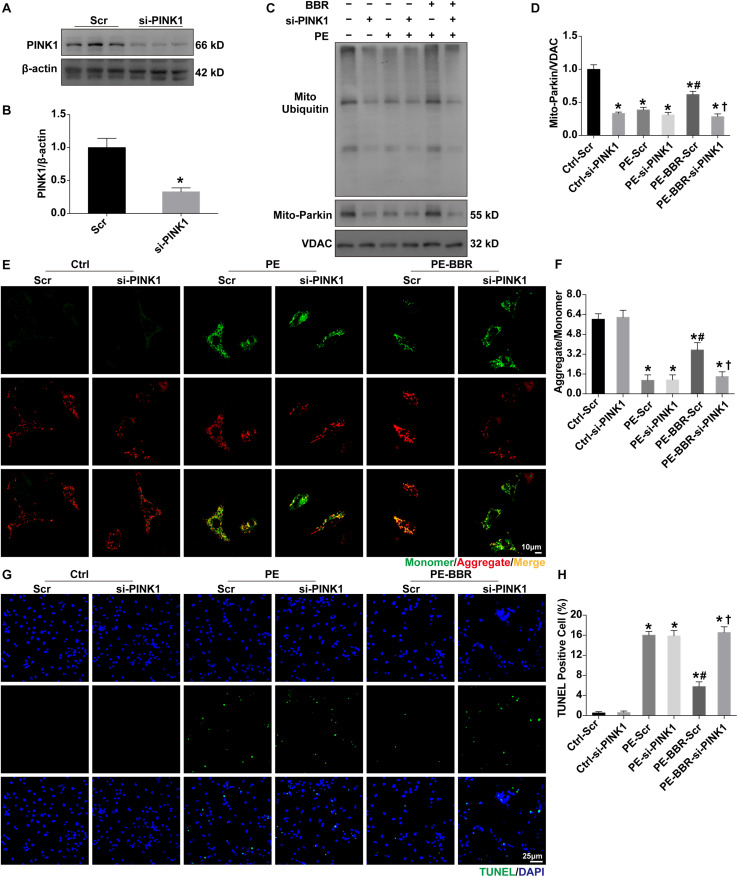
Effects of PINK1 knockdown on BBR-induced benefits in NMCMs treated with or without PE. **(A,B)** Expression of PINK1 in presence or absence of si-PINK1 (normalized to β-actin) (*n* = 3); **(C)** Representative gel blots of mitochondrial ubiquitin and Parkin for each group; **(D)** Mito- Parkin to VDAC ratio (*n* = 3); **(E)** Representative JC-1 staining images of mitochondrial membrane potential (MMP) for each group (Scale bar = 10 μm); **(F)** Quantified analysis of MMP for each group (MMP as ratio of the red fluorescence obtained at 590 nm to the green fluorescence at 530 nm) (*n* = 6); **(G)** Representative TUNEL/DAPI staining images for each group (Scale bar = 25 μm); **(H)** Quantified data of cardiomyocytes apoptosis for each group (*n* = 6). Mean ± SEM, * *p* < 0.05 vs. Ctrl-Scr group; # *p* < 0.05 vs. PE-Scr group, † *p* < 0.05 vs. PE-BBR-Scr group.

In order to confirm the role of PINK1 in the regulation of berberine on mitophagy, western blot was used to examine the changes of mitochondrial Parkin, ubiquitin and p62. Firstly, the protein expression of PINK1 has been confirmed to be downregulated with the presence of si-PINK1 (Figures 6A,B). Then we observed that berberine administration significantly increased levels of Parkin, ubiquitin and p62 in PE-treated cells, whereas these increases were blunted through downregulation of PINK1 (Figures 6C,D). JC-1 staining revealed that the addition of si-PINK1 failed to restore mitochondrial membrane potential in PE-BBR cells (Figures 6E,F). Furthermore, reduction of apoptosis cells assessed by TUNEL assays was observed with berberine after the stimulation of PE, the effect of which was also diminished by si-PINK1 (Figures 6G,H). Taken together, these data suggest that berberine attenuates the development of TAC induced HF by stimulating PINK1/Parkin/Ubiquitin-dependent activation of mitochondrial autophagy activity.

## Discussion

The marked findings from our present study demonstrated that PINK1/Parkin/Ubiquitin-dependent mitophagy was downregulated both in myocardium of HF induced by TAC-elicited pressure overload *in vivo* and in cardiomyocytes treated with phenylephedrine i*n vitro*. Overt dysfunctional mitochondria, apoptotic cardiomyocytes and decreased cardiac function were concurrently observed along with the depressed mitophagy. Berberine administration activated PINK1/Parkin/Ubiquitin-dependent mitophagy, accelerated the clearance of damaged mitochondria, rescued the injured cardiomyocytes and partially restored cardiac function. Our findings suggested that PINK1/Parkin/Ubiquitin mechanism may be involved in berberine mediated cardioprotective effects on TAC-induced adverse cardiac hypertrophy, and even on chronic HF.

During cardiomyocyte injury and left ventricular remodeling, berberine was found to have distinct functions: berberine could play protective role during ischemia or hypoxia, whereas it may be deleterious to the heart. Previous studies shown that berberine has effects of attenuating cardiac remodeling and cardiomyocyte apoptosis. For example, *Zhu et al.* demonstrated that berberine protects cells from hypoxia-induced apoptosis through inhibiting of autophagy and suppression of AMPK activation (Jia et al., 2017). Pretreatment with berberine via oral administration, improved MI/R injury by activating the signaling pathway of AK2/STAT3 and attenuating cell apoptosis. In addition, the protective effect of berberine *in vitro* and *in vivo*, was demonstrated in model of HF (Zhao et al., 2016). After myocardial infarction, berberine ameliorated the cardiac remodeling and inhibited cardiac dysfunction in HF. Its mechanism may be upregulating Bcl-2/Bax and down-regulating caspase-3 expression (Liao et al., 2018). Notably, in rat model of cardiac hypertrophy berberine could attenuate left ventricular remodeling and cardiomyocyte apoptosis through an autophagy-dependent mechanism via inhibition of mTOR, p38 and ERK1/2 MAPK signaling pathways (Li et al., 2014). In our study, TAC induced significant cardiomyocytes apoptosis, cardiac hypertrophy, interstitial fibrosis and cardiac dysfunction combined with suppressed mitophagy and accumulation of damaged mitochondria.

Coincide with the findings described above, we also proved the anti-apoptosis and autophagy regulation characteristics of berberine in TAC. Autophagy is activated to promote cell survival by releasing energy substrates via degradation of cellular constituents (Lavandero et al., 2015). Berberine improves pressure overload-induced cardiac hypertrophy and dysfunction through enhanced autophagy (Li et al., 2014). A great deal of data demonstrates that excessive autophagy elicited by pathological stimuli, such as pressure overload or I/R myocardial injury, is maladaptive and promotes cell apoptosis. Excessive activation of autophagy has been reported to lead to programmed cell death, aggravate cardiac hypertrophy and speed up the process of HF (Kheloufi et al., 2017).

Mitophagy is a selective autophagy which promotes the specific engulf of damaged mitochondria targeting to lysosomes for degradation (Marín-García and Akhmedov, 2016; Vásquez-Trincado et al., 2016). In our study, berberine also activated mitophagy activity as evidenced by elevated mitochondrial p62 and decreased LC3II. Additionally, berberine could increase number of mitochondrial autophagosomes which then promote the clearance of damaged mitochondria, maintained mitochondrial hemostasis and preserved cardiac function. These results indicated that mitophagy regulation is involved in berberine-mediated beneficial effects on TAC-induced HF.

The mechanism(s) involved in mitophagy regulation include PINK1/Parkin- and mitophagy receptors-dependent pathway (Eiyama and Okamoto, 2015). The study showed that PINK1/Parkin acts cooperatively in adjusting mitochondrial balancing function and marking impaired mitochondria (Fan et al., 2019; Wen et al., 2020). Notably, ubiquitylation and deubiquitylation play roles in modulating significantly (Tanaka, 2020). Our findings revealed that TAC induced downregulation of PINK1/Parkin/Ubiquitin pathway while not influenced the level of FUNDC1, PHB2 and BNIP3L, which are the most studied receptors of mitophagy regulation. When treated with berberine, PINK/Parkin/ubiquitin signaling cascade was activated without changes of FUNDC1, PHB2 and BNIP3L. Knockdown of PINK1 by si-RNA significantly suppressed mitochondrial translocation of Parkin and subsequent ubiquitination of mitochondrial out membrane proteins. PINK1/Parkin pathway was proved to participate in pathological cardiac hypertrophy and HF (Fan et al., 2019). PINK1 knockout mice develop cardiac hypertrophy and left ventricular dysfunction by 2 months of age, which was associated with increased oxidative stress and mitochondrial dysfunction (Billia et al., 2011). Phosphorylation of Ser495 in PINK1 by AMPKα2 is essential for efficient mitophagy to prevent the progression of HF (Wang et al., 2018). Consistently, our study revealed the critical role of PTEN-induced putative kinase 1-dependent mitophagy in the development of HF.

In conclusion, we identified berberine can protect cardiac function from HF through augmented mitophagy via the cooperative role of PINK1 and Parkin, which then provides a possible clinical strategy for the treatment of HF. However, more work should be taken to investigate other mechanism(s) of berberine on HF and find new targets for the clinical management.

## Data Availability Statement

The raw data supporting the conclusions of this article will be made available by the authors, without undue reservation.

## Ethics Statement

The animal study was reviewed and approved by The Ethics committee of Shanghai Xuhui Central Hospital/Zhongshan-Xuhui Hospital, Fudan University.

## Author Contributions

MA did the evolution of overarching research goals and aims and specifically writing the initial draft. WY did the design of methodology and specifically critical review. RC did the presentation of the published work. YZ did the leadership responsibility for the research activity planning and execution. HZ did the project administration and funding acquisition. All authors contributed to the article and approved the submitted version.

## Conflict of Interest

The authors declare that the research was conducted in the absence of any commercial or financial relationships that could be construed as a potential conflict of interest.

## Supplementary Material

Supplementary Figure 1 Kaplan-Meier curves of mice with berberine administration at five different doses. **(A)** Doses (0, 5, 7.5, 10, 20 mg/kg) of berberine per day for intraperitoneal injection; **(B)** Doses (0, 10, 50, 75, 100 mg/kg) of berberine per day for oral administration group. *n* = 8 mice per group.

Supplementary Figure 2 Echocardiographic properties of mice with berberine administrated at different time points (1 day, 3 days, 1 week, 2 weeks) after TAC surgery for 4 weeks. **(A)** Experimental design; **(B–Q)** Quantified analysis of indexes included left ventricular ejection fraction (LVEF), fractional shortening (FS), left ventricular end diastolic volume (LVEDV) and left ventricular end systolic volume (LVESV) (*n* = 5). Mean ± SEM, * *p* < 0.05 vs. Sham group; # *p* < 0.05 vs. TAC group.
