# Supplementary figures and images for: Berberine Promotes Cardiac Function by Upregulating PINK1/Parkin-Mediated Mitophagy in Heart Failure
Source: Front Physiol. 2020 Sep 25;11:565751. doi: 10.3389/fphys.2020.565751 (PMC7546405; doi:10.3389/fphys.2020.565751)

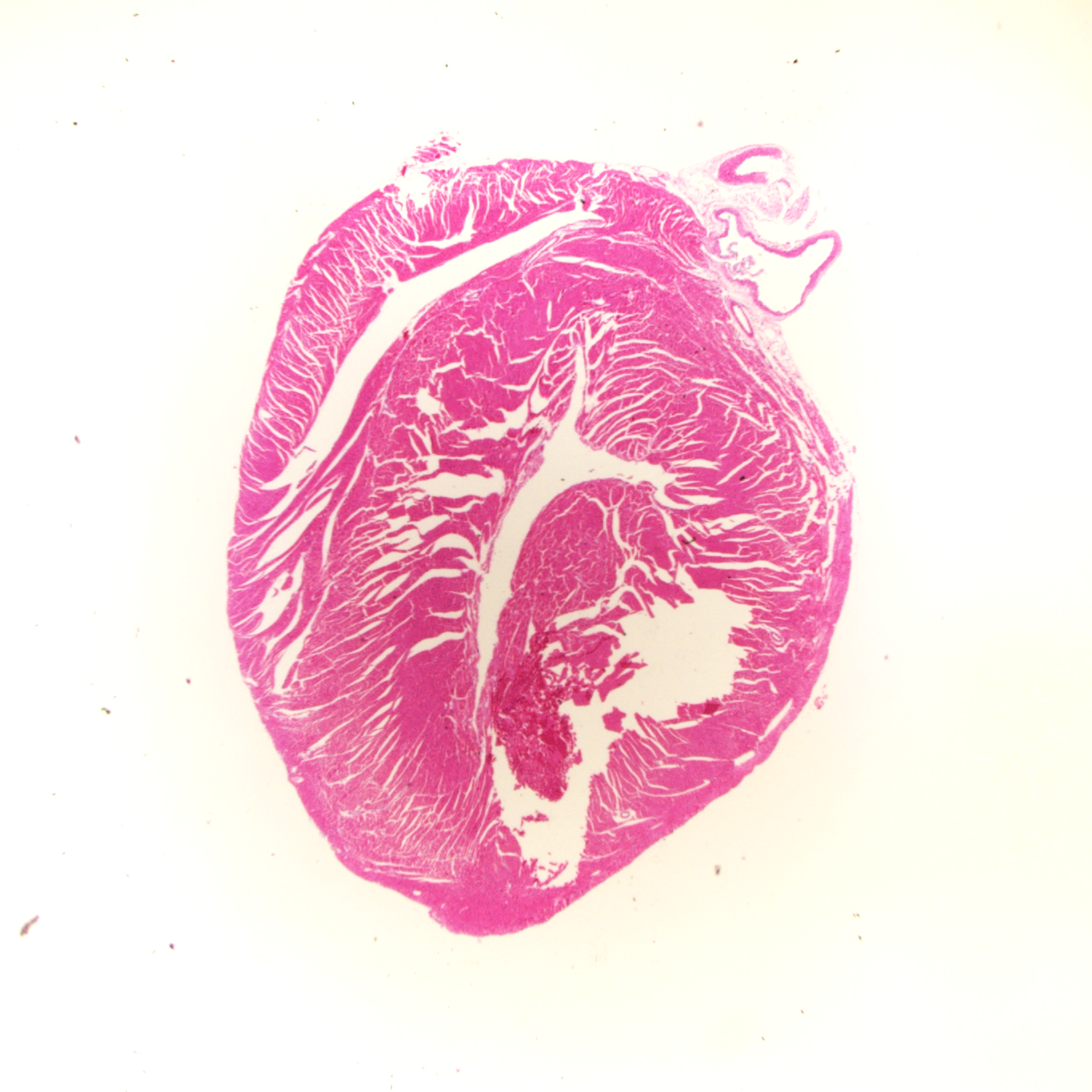

Supplement: Supplementary Figure 1 — Kaplan-Meier curves of mice with berberine administration at five different doses. (A) Doses (0, 5, 7.5, 10, 20 mg/kg) of berberine per day for intraperitoneal injection; (B) Doses (0, 10, 50, 75, 100 mg/kg) of berberine per day for oral administration group. n = 8 mice per group. [file Image_1.TIF]

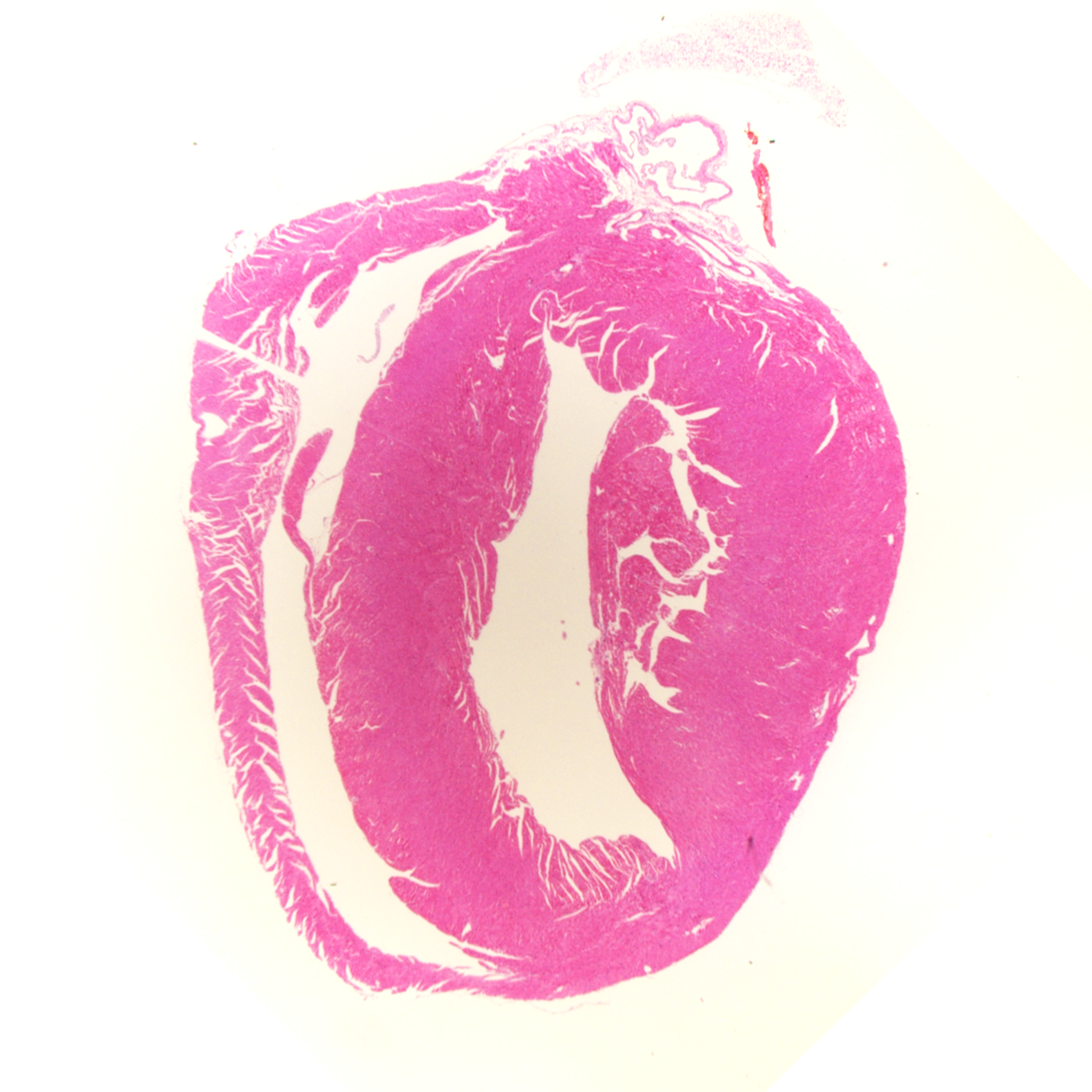

Supplement: Supplementary Figure 2 — Echocardiographic properties of mice with berberine administrated at different time points (1 day, 3 days, 1 week, 2 weeks) after TAC surgery for 4 weeks. (A) Experimental design; (B–Q) Quantified analysis of indexes included left ventricular ejection fraction (LVEF), fractional shortening (FS), left ventricular end diastolic volume (LVEDV) and left ventricular end systolic volume (LVESV) (n = 5). Mean ± SEM, * p < 0.05 vs. Sham group; # p < 0.05 vs. TAC group. [file Image_2.TIF]

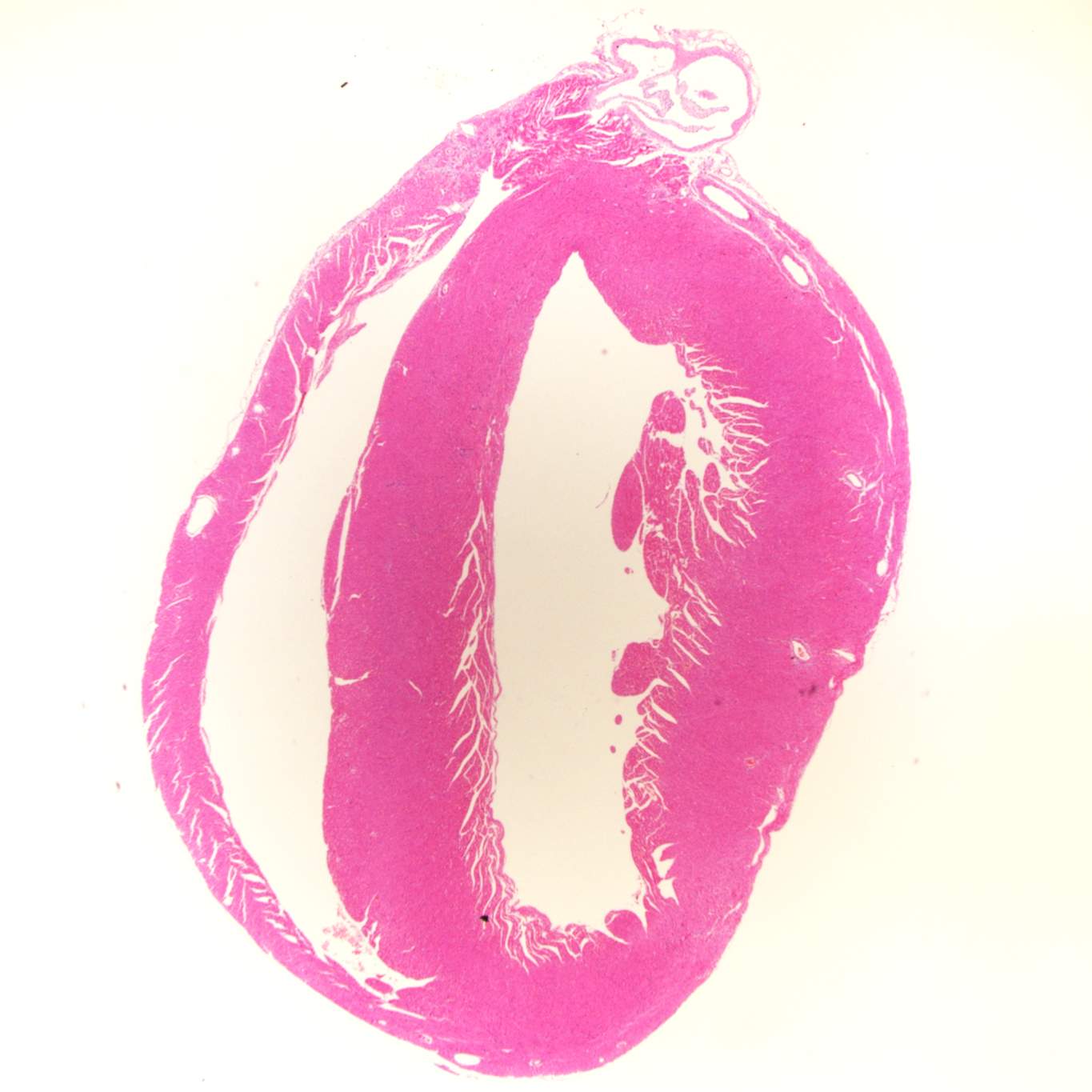

Supplement: Supplementary file 3 [file Image_3.TIF]

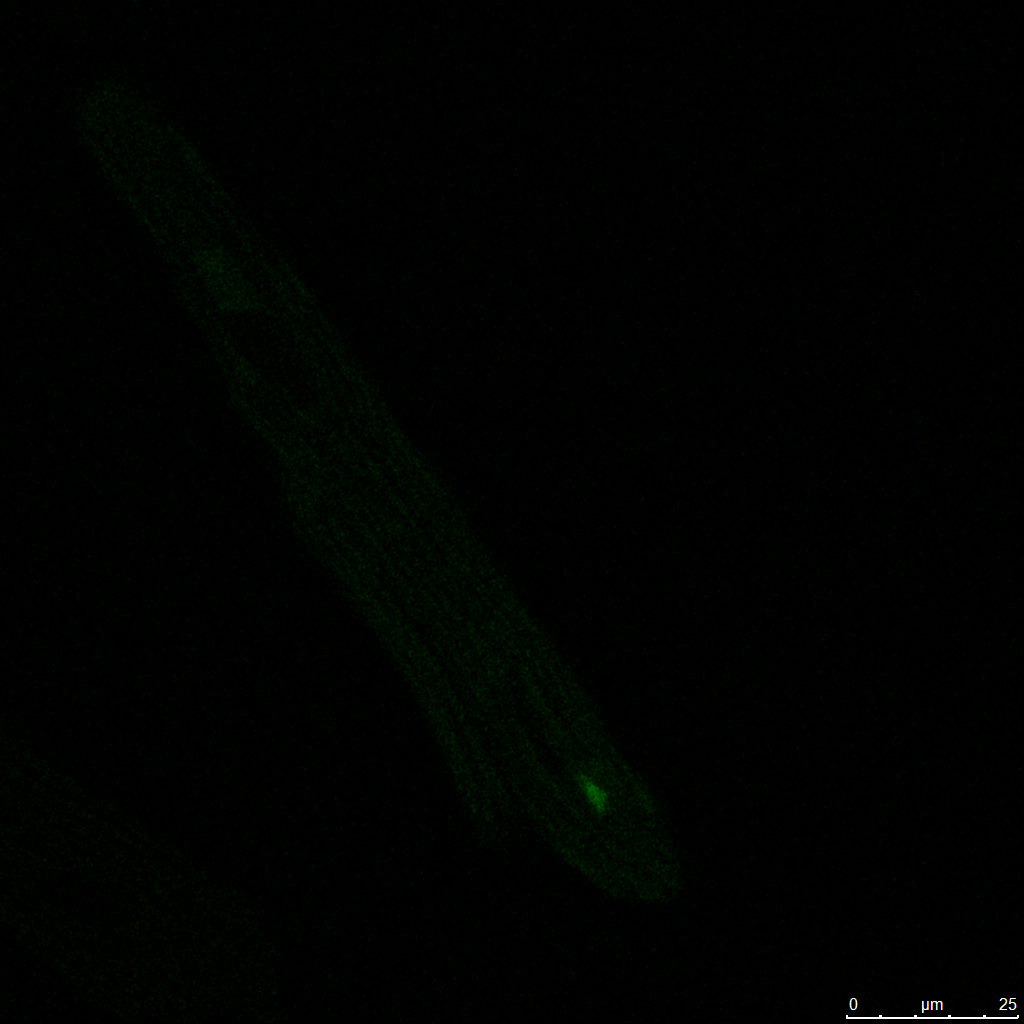

Supplement: Supplementary file 4 [file Image_4.TIF]

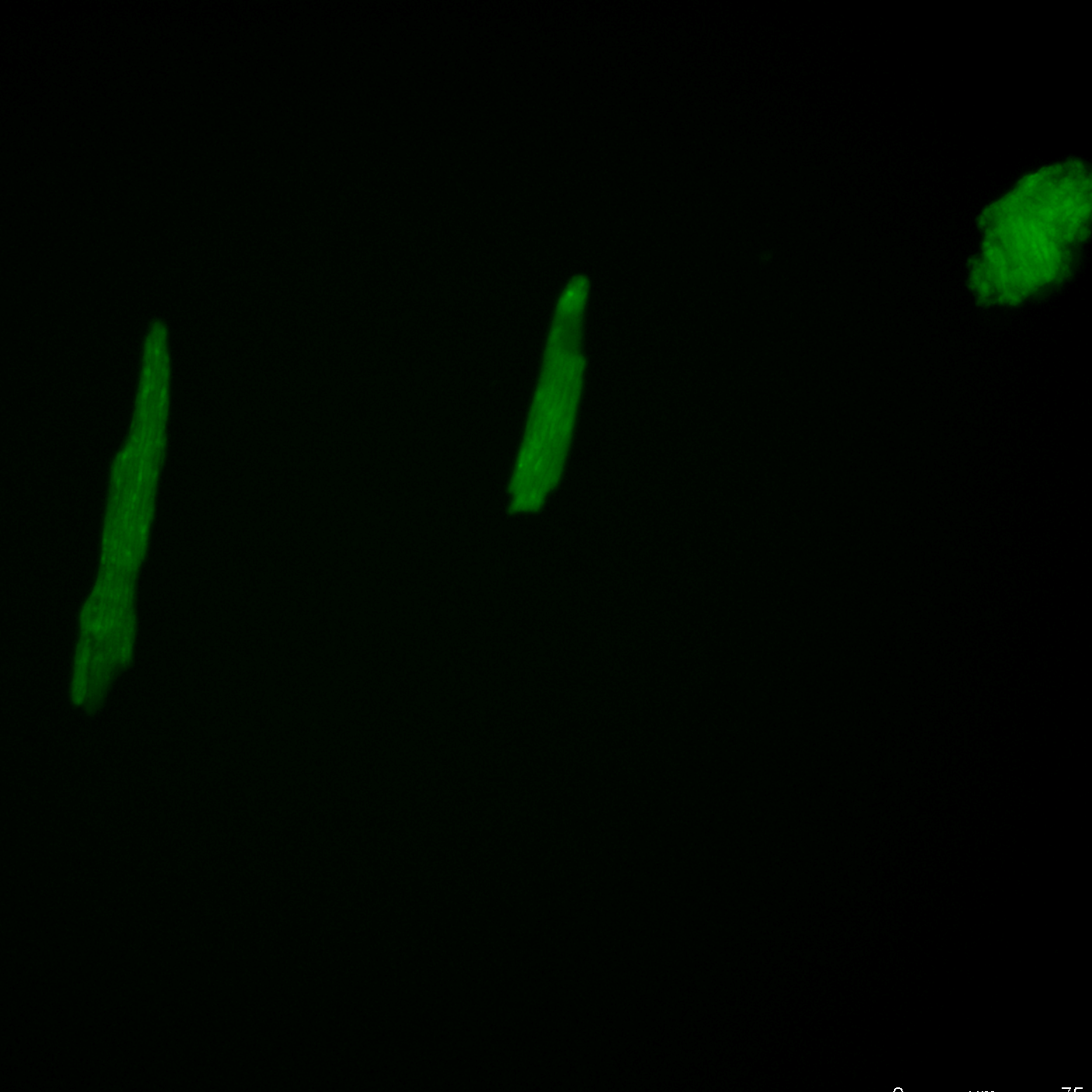

Supplement: Supplementary file 5 [file Image_5.TIF]

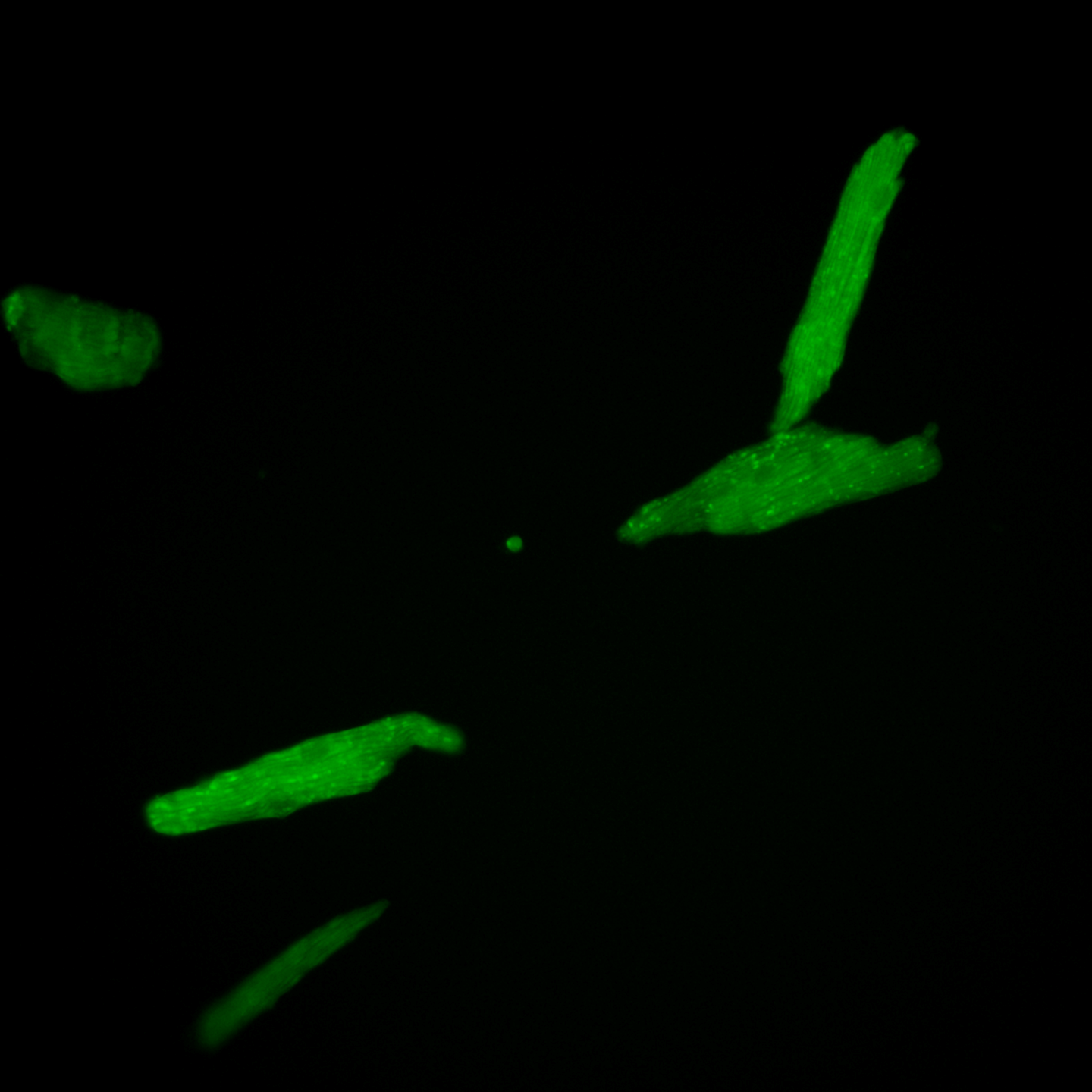

Supplement: Supplementary file 6 [file Image_6.TIF]

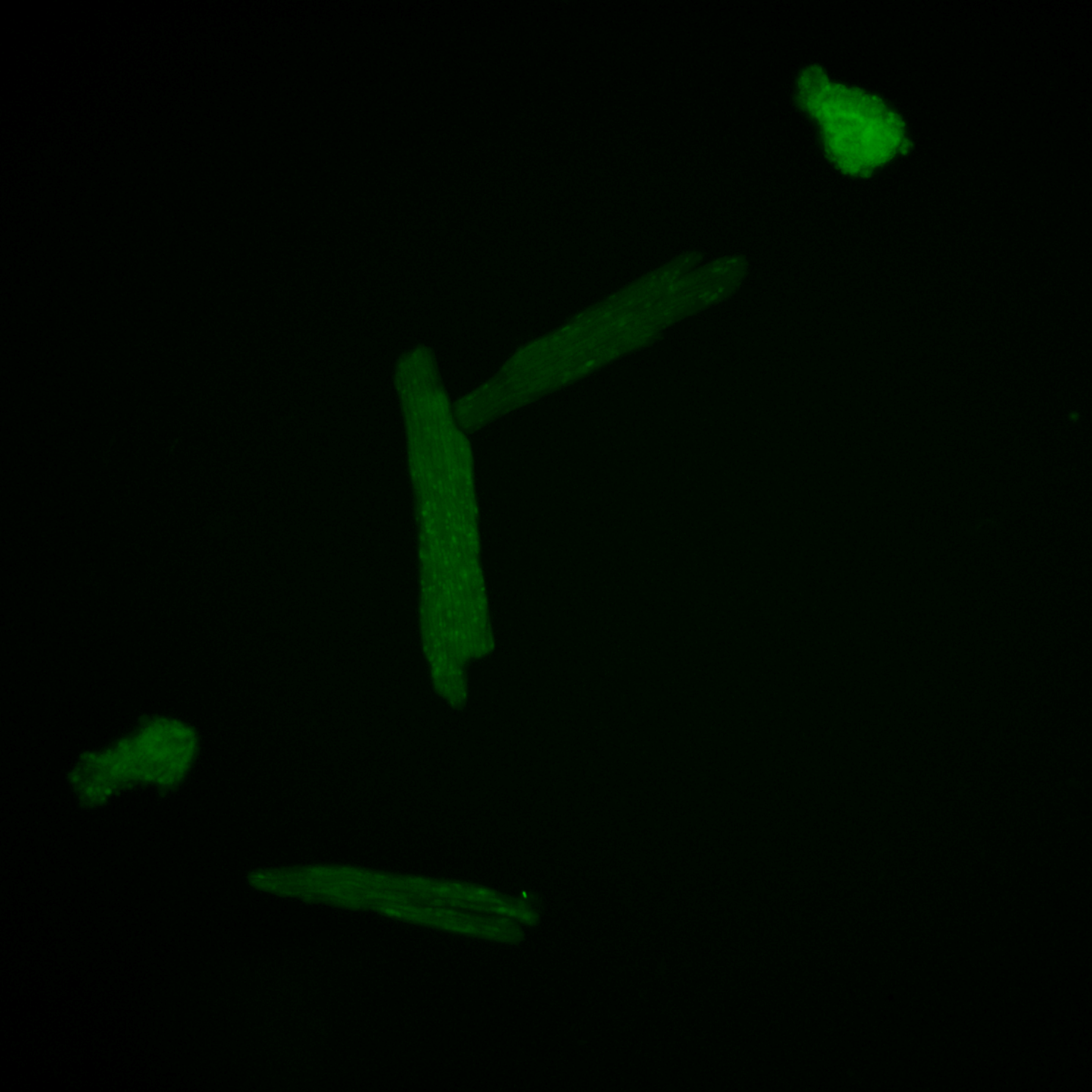

Supplement: Supplementary file 7 [file Image_7.TIF]

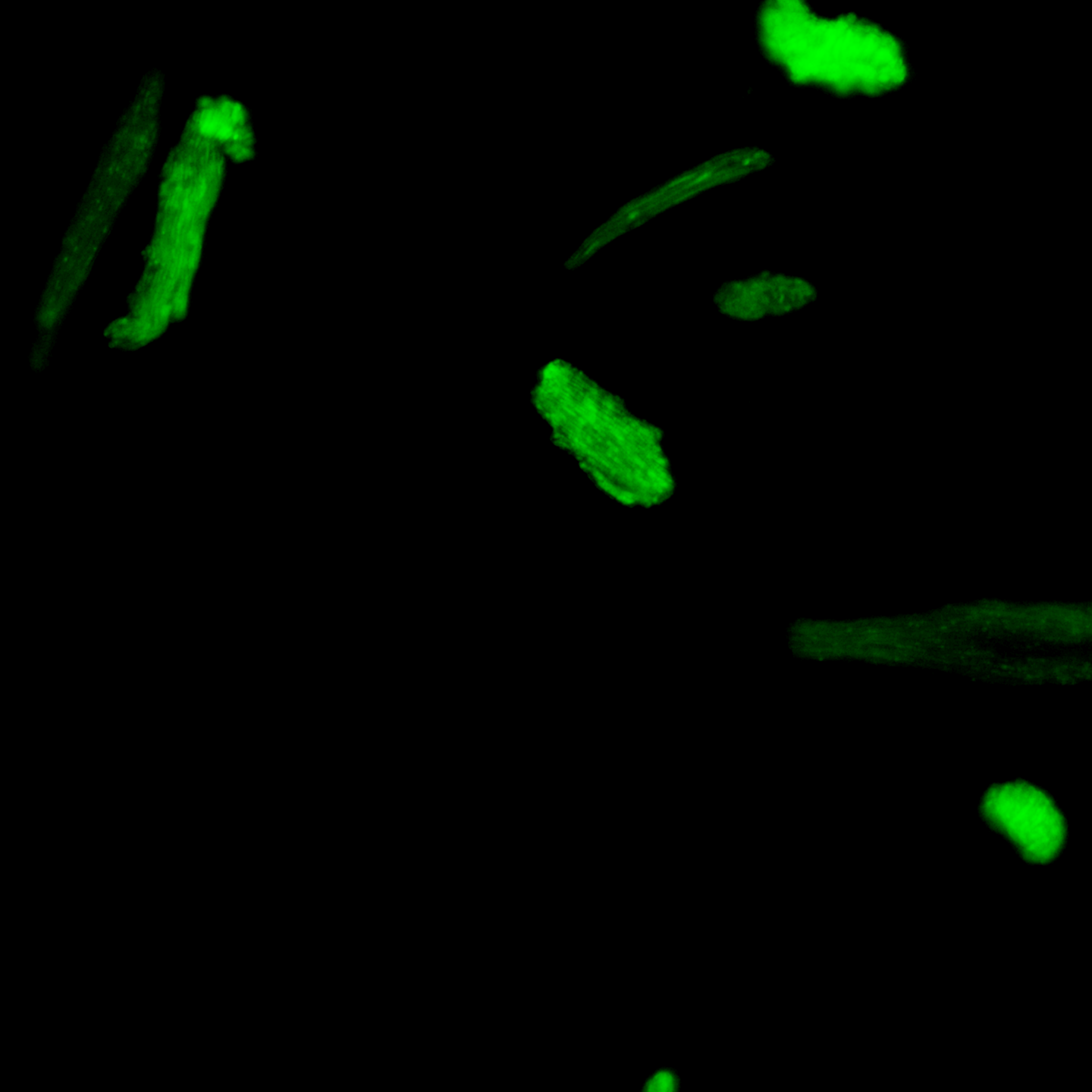

Supplement: Supplementary file 8 [file Image_8.TIF]

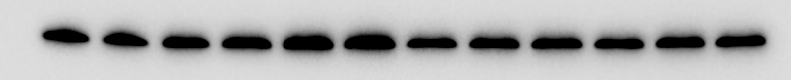

Supplement: Supplementary file 9 [file Image_9.TIF]

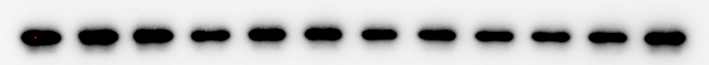

Supplement: Supplementary file 10 [file Image_10.TIF]

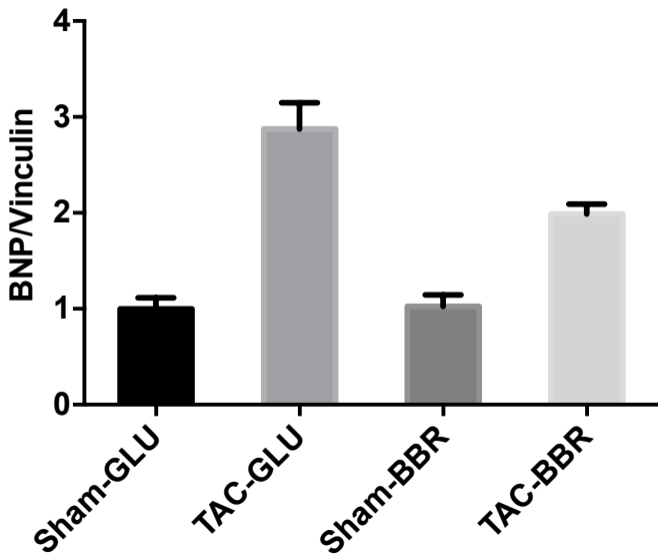

Supplement: Supplementary file 11 [file Data_Sheet_1.PDF]

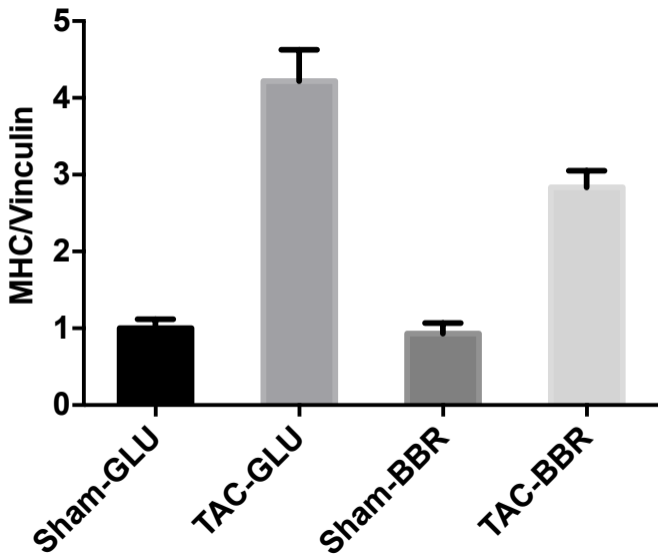

Supplement: Supplementary file 12 [file Data_Sheet_2.PDF]

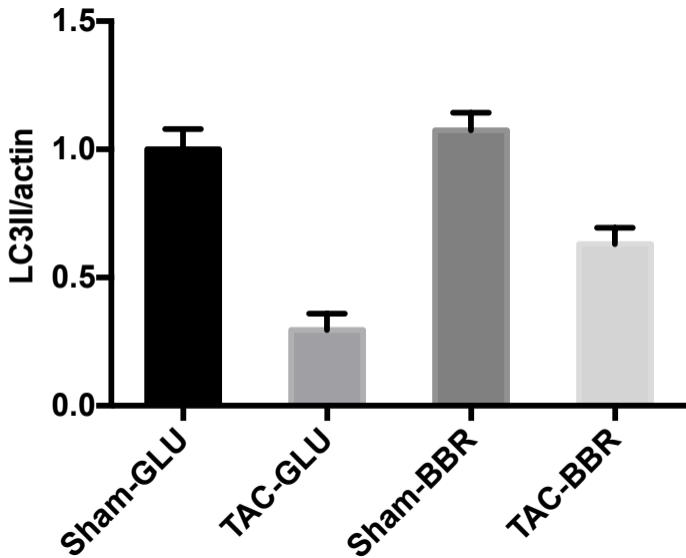

Supplement: Supplementary file 13 [file Data_Sheet_3.PDF]

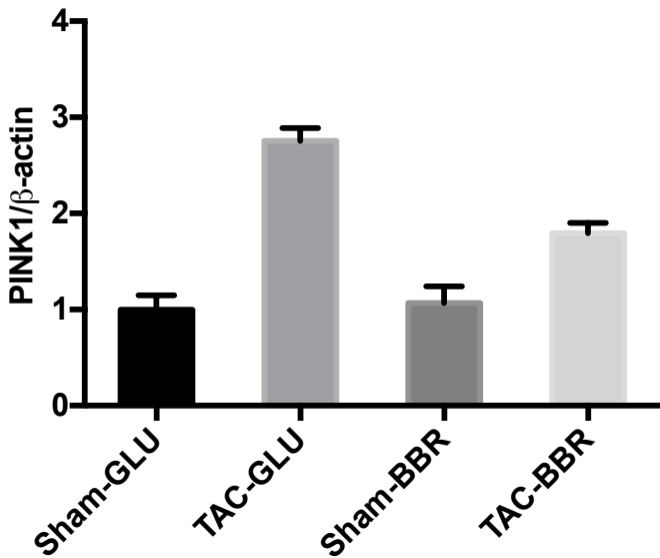

Supplement: Supplementary file 14 [file Data_Sheet_4.PDF]

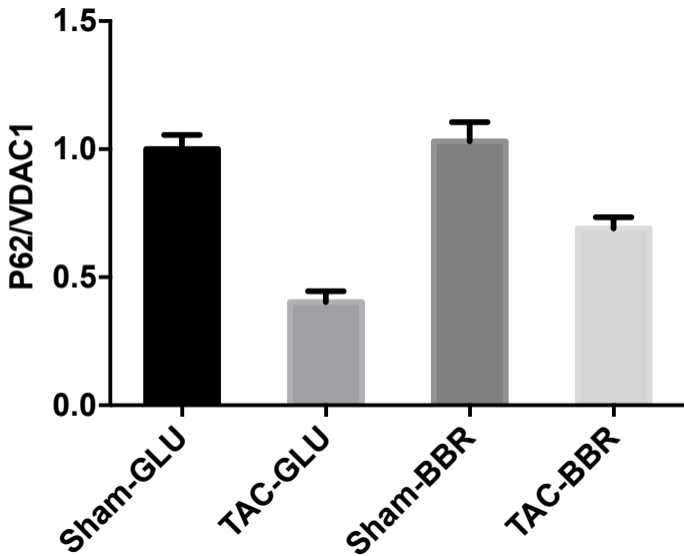

Supplement: Supplementary file 15 [file Data_Sheet_5.PDF]

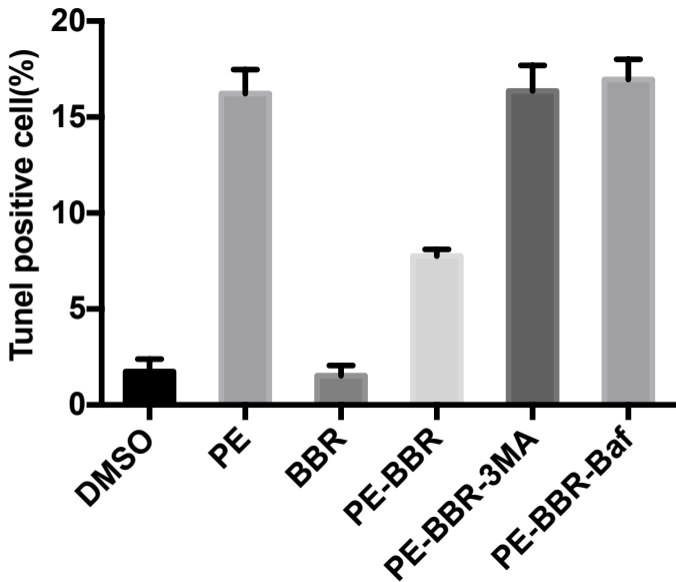

Supplement: Supplementary file 16 [file Data_Sheet_6.PDF]

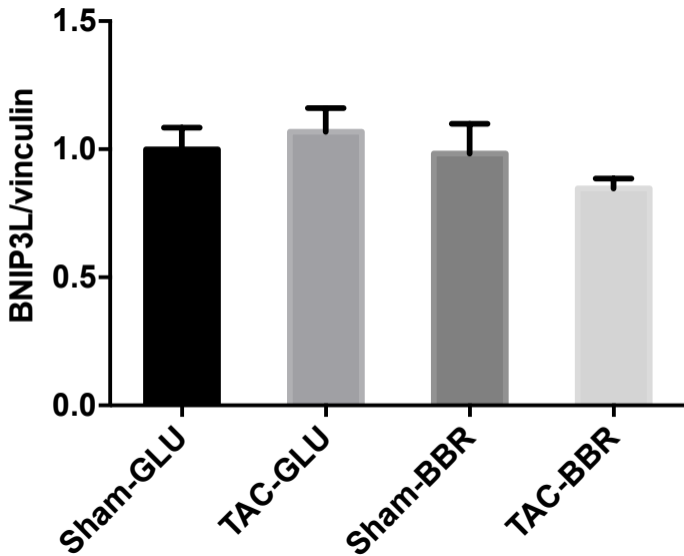

Supplement: Supplementary file 17 [file Data_Sheet_7.PDF]

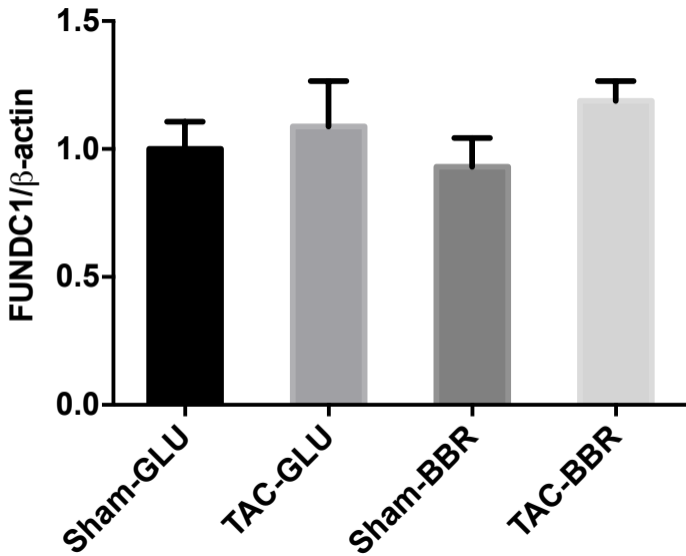

Supplement: Supplementary file 18 [file Data_Sheet_8.PDF]

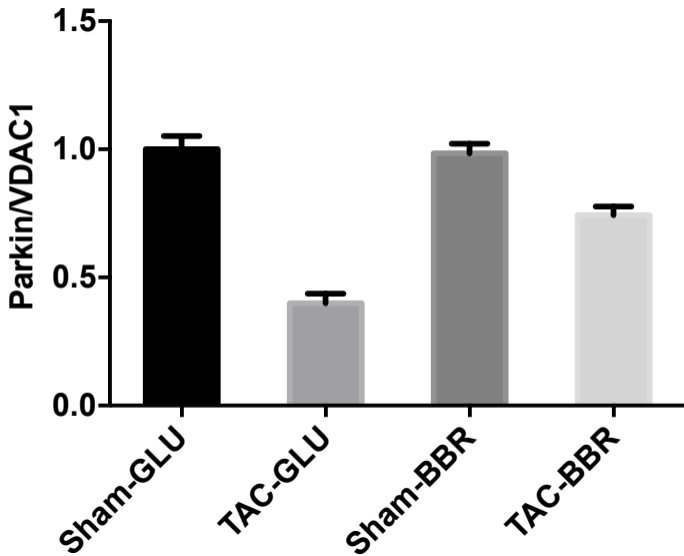

Supplement: Supplementary file 19 [file Data_Sheet_9.PDF]

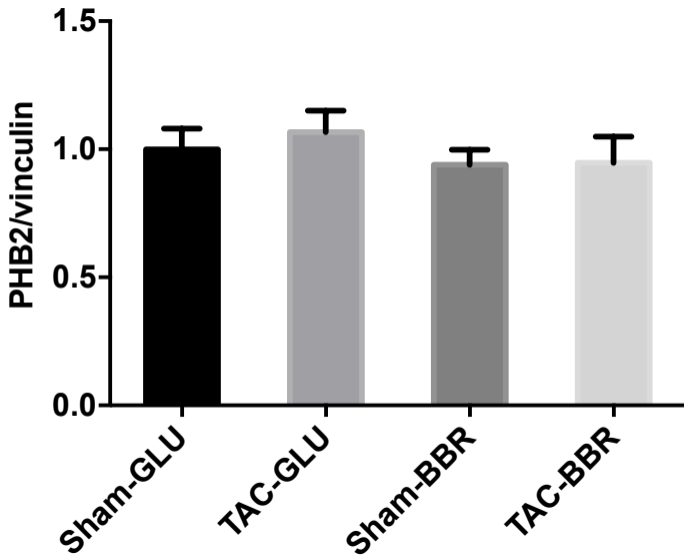

Supplement: Supplementary file 20 [file Data_Sheet_10.PDF]

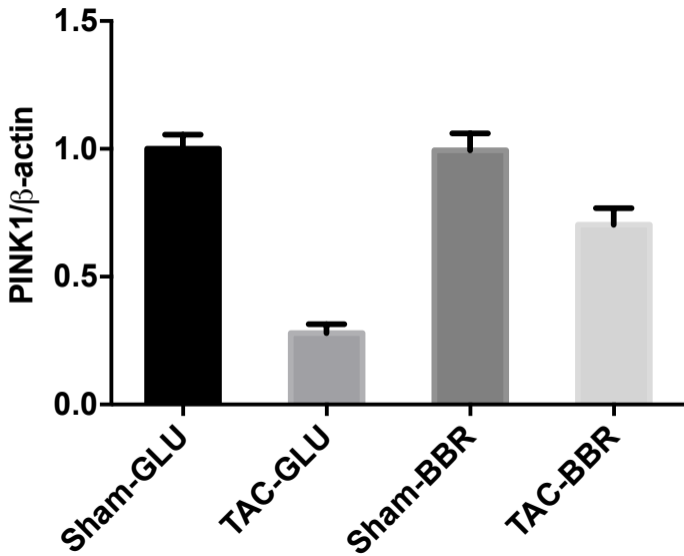

Supplement: Supplementary file 21 [file Data_Sheet_11.PDF]

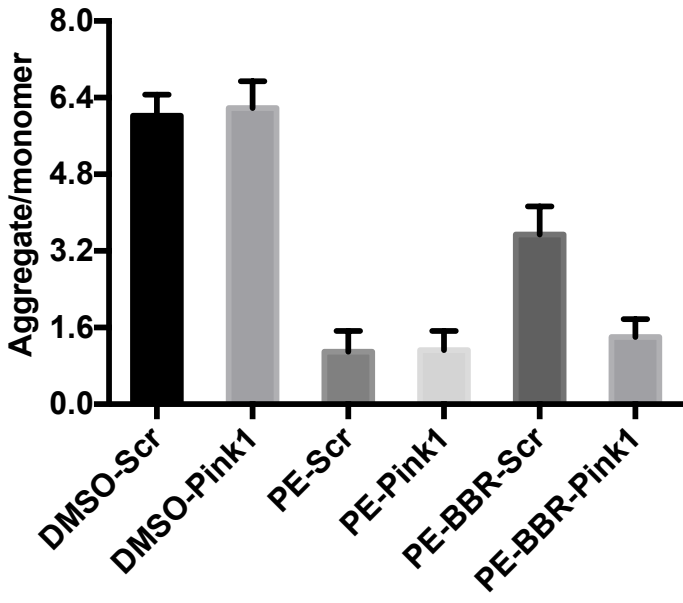

Supplement: Supplementary file 22 [file Data_Sheet_12.PDF]

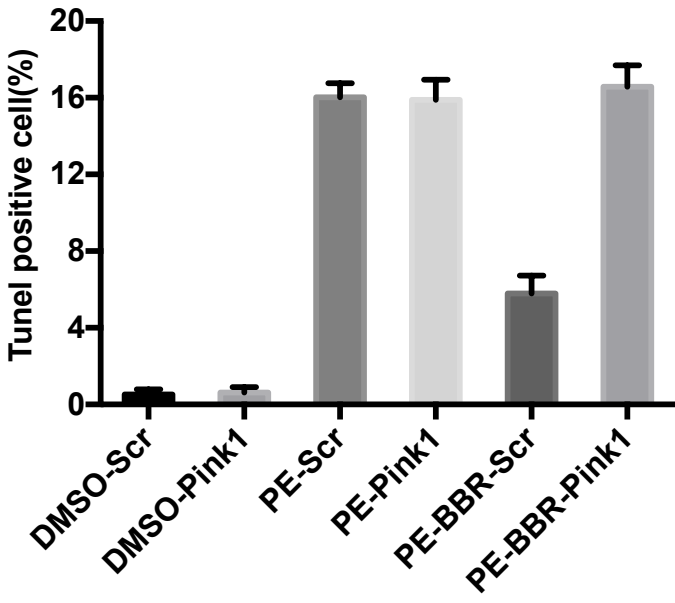

Supplement: Supplementary file 23 [file Data_Sheet_13.PDF]

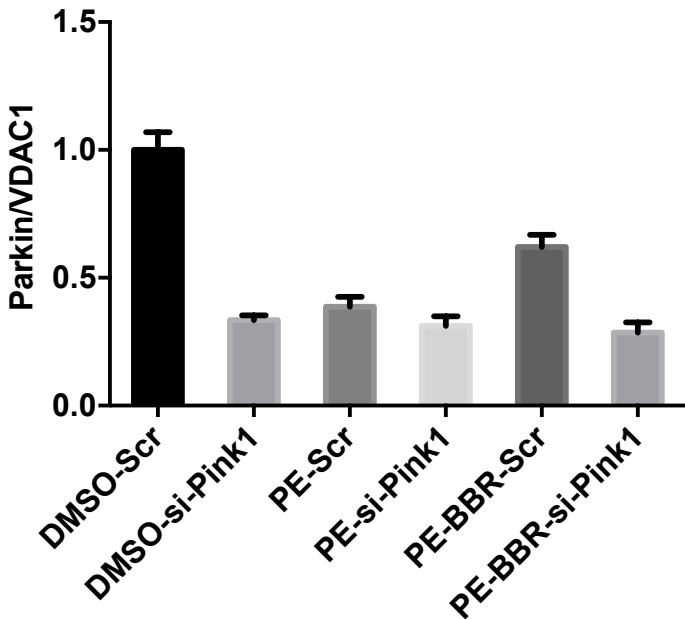

Supplement: Supplementary file 24 [file Data_Sheet_14.PDF]

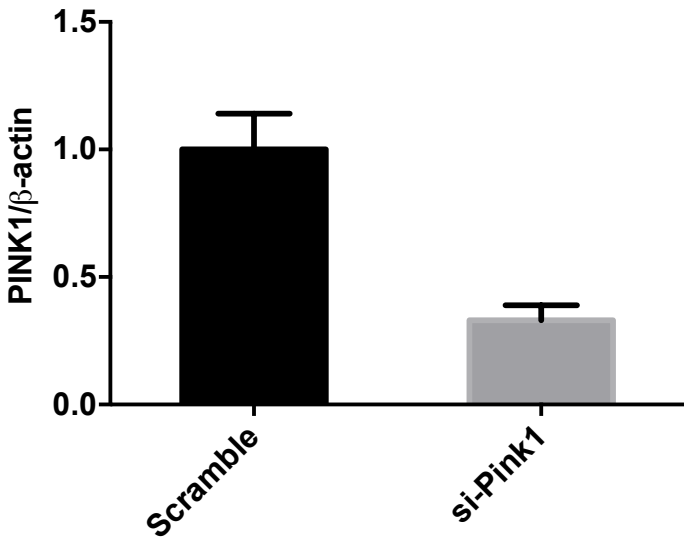

Supplement: Supplementary file 25 [file Data_Sheet_15.PDF]
